# Styloid process morphology in extracranial internal carotid artery dissection: a CTA case-control study

**DOI:** 10.1186/s12880-026-02415-y

**Published:** 2026-05-11

**Authors:** Xueke Zhang, Xin Shen, Shaokun Hu, Yiru Shen, Zeyuan Cao, Yuanyuan Wu, Dongliang Hu, Manman Cui, Duchang Zhai, Dai Shi, Wu Cai, Shenghong Ju

**Affiliations:** 1 Department of Radiology, The Second Affiliated Hospital of Soochow University, San Xiang Road No. 1055, Suzhou, Jiangsu 215004 China; 2 Suzhou Medical College of Soochow University, 199 Ren’ai Road, Huqiu District, Suzhou, Jiangsu 215026 China; 3 Department of Radiology, Zhongda Hospital, Medical School of Southeast University, Ding Jia Qiao Road No. 87, Nanjing, Jiangsu 210009 China

**Keywords:** Styloid process, Internal carotid artery dissection, CTA, Eagle syndrome

## Abstract

**Background:**

To evaluate the association between styloid process (SP) morphology derived from CTA and extracranial internal carotid artery dissection (ICAD).

**Materials and methods:**

This retrospective case-control study included 85 patients with unilateral extracranial ICAD and 85 frequency-matched controls who underwent head and neck CTA. Two neuroradiologists independently measured SP length, the distance from the SP tip to the internal carotid artery (SPT–ICA distance), and SP orientation (medial and anterior inclination angles). Group comparisons were performed between the dissection side in the ICAD group and the corresponding matched side in controls. Multivariable logistic regression was used to identify independent morphologic factors associated with ICAD, and ROC analysis was performed to evaluate the diagnostic performance of each individual parameter and of the combined model.

**Results:**

Compared with controls, ICAD showed a longer SP, a shorter SPT–ICA distance, and a smaller anterior inclination angle (all *p* < 0.001). Multivariable logistic regression identified SP length, SPT–ICA distance, and anterior inclination angle as independent factors associated with ICAD. ROC analysis showed that the combined model achieved the best performance (AUC = 0.836), compared with SP length (AUC = 0.752), SPT–ICA distance (AUC = 0.742), and anterior inclination angle (AUC = 0.717).

**Conclusion:**

The combined model based on SP length, SPT–ICA distance, and anterior inclination angle showed the best performance, suggesting that these CTA-derived SP morphologic features may help identify an anatomic pattern associated with extracranial ICAD.

## Introduction

Carotid artery dissection is a leading cause of ischemic stroke in young adults [1]. Its pathophysiology involves the interplay between arterial wall abnormalities and external triggers. Intrinsic vulnerabilities may include fibromuscular dysplasia [2], increased arterial stiffness [3], connective tissue abnormalities [4], or genetic predisposition [5], whereas extrinsic triggers often involve neck hyperextension or rotation, coughing, or minor trauma [6]. These observations provide clues to the causes of so-called “spontaneous” dissections, but the specific triggering mechanisms remain incompletely understood [7]. In particular, ischemic stroke secondary to styloid process (SP)–related internal carotid artery dissection (ICAD) has gained growing clinical recognition, yet the anatomic determinants and high-risk morphologic features remain poorly defined.

The SP is a thin bony projection extending downward and anteromedially from the base of the temporal bone. Its tip usually lies near the carotid bifurcation, between the internal and external carotid arteries. Together with the stylohyoid ligament and the lesser horn of the hyoid bone, it forms the stylohyoid complex [8]. In 1937, Eagle first described “Eagle syndrome”, referring to symptoms caused by an elongated SP or ossification of the stylohyoid ligament. Depending on which structures are involved, the symptoms can be classified as neurogenic or vascular [9]. Neurogenic symptoms include head and neck pain, hoarseness, and dysphagia, resulting from compression or traction of the caudal branches of the cranial nerves by the SP. Vascular symptoms result from compression or injury of adjacent vessels, leading to stenosis or dissection and subsequent transient ischemic attack or cerebral infarction [10, 11]. Approximately 4% of the general population have an elongated SP, but only 4–10% develop related clinical symptoms [12]. This suggests that “an elongated SP” alone is insufficient to explain all clinical events, and that its spatial relationship with the surrounding vessels is more critical. To date, comprehensive evaluations of multiple SP morphologic features in the context of ICAD are limited, and it remains uncertain which geometric features contribute most.

Based on this background, we assessed three morphological features of the SP: the length of SP, the distance from the SP tip to the internal carotid artery (SPT–ICA distance) and directional orientation (medial and anterior inclination angles), and examined their associations with extracranial ICAD. We further evaluated whether SP elongation and closer SPT–ICA proximity, reflected by both distance and angles, are linked to a higher likelihood of ICAD, to inform clinical risk recognition and structured radiology reporting.

## Methods

### Patient selection

This retrospective case–control study was approved by our institutional ethics committee. The requirement for written informed consent was waived due to the retrospective design and the use of de-identified clinical and imaging data.

**Fig. 1:**
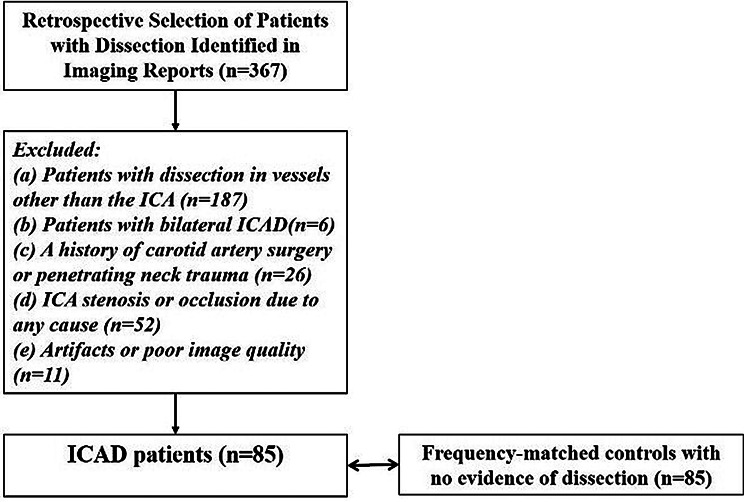
The patient recruitment process

We searched the radiology database of our institution for head and neck CTA reports from 2017 to 2024 using “dissection” as the keyword for initial screening. Two radiologists independently reviewed the reports and source images to confirm unilateral extracranial ICAD based on standard CTA features. Disagreements were resolved by consensus. Exclusion criteria were as follows: (1) a history of carotid artery surgery or penetrating neck trauma; (2) carotid artery stenosis or occlusion unrelated to dissection (e.g., atherosclerotic stenosis or thromboembolic occlusion, etc.); (3) artifacts or poor image quality. Controls were selected during the same period to achieve a similar distribution of age, sex, and laterality as the ICAD group. Control CTA examinations were performed for clinical indications such as headache, dizziness, or suspected cerebrovascular disease, but were required to have no imaging evidence of dissection and no history of carotid surgery, penetrating neck trauma, or carotid occlusion (Fig. 1) [13].

### CTA acquisition protocol

All head and neck CTA examinations were performed on an IQon spectral CT scanner (Philips Healthcare, the Netherlands). Patients were examined in the supine position with head-first positioning. All examinations were performed according to the institutional head-and-neck CTA protocol, with routine positioning aimed at minimizing head rotation and tilt during acquisition. The scan range covered the region from the aortic arch to the vertex. The acquisition parameters were as follows: section thickness, 0.9 mm; reconstruction interval, 0.7 mm; tube voltage, 120 kV; tube current, 252 mA; detector collimation, 128 × 0.625 mm; pitch, 0.992; gantry rotation time, 0.5 s/rotation; and matrix size, 512 × 512. Contrast-enhanced CTA was performed by injecting iodinated contrast material through an antecubital vein at a dose of 0.8 mL/kg and a flow rate of 5 mL/s, followed by a 30 mL saline flush at the same injection rate.

### Image reconstruction and measurement definitions

Measurements were performed by two neuroradiologists independently. The original CTA images were transferred to an IntelliSpace Portal workstation (Philips Healthcare, the Netherlands), and post-processing was performed using maximum intensity projection (MIP) and multi-planar reconstruction (MPR). In this study, we followed previously published CTA-based measurement methods for SP length and orientation with minor modifications according to our institutional imaging workflow [12, 14]. The orbitomeatal line (OML) and the line connecting the midpoints of the bilateral SP bases were used as horizontal baselines to improve measurement reproducibility [14, 15]. Before angle measurement, multiplanar reformations were adjusted using reproducible craniofacial bony landmarks, including the orbital margin and the external auditory canal, to allow consistent identification of the OML as the horizontal baseline.

#### The length of SP (mm)

**Fig. 2:**
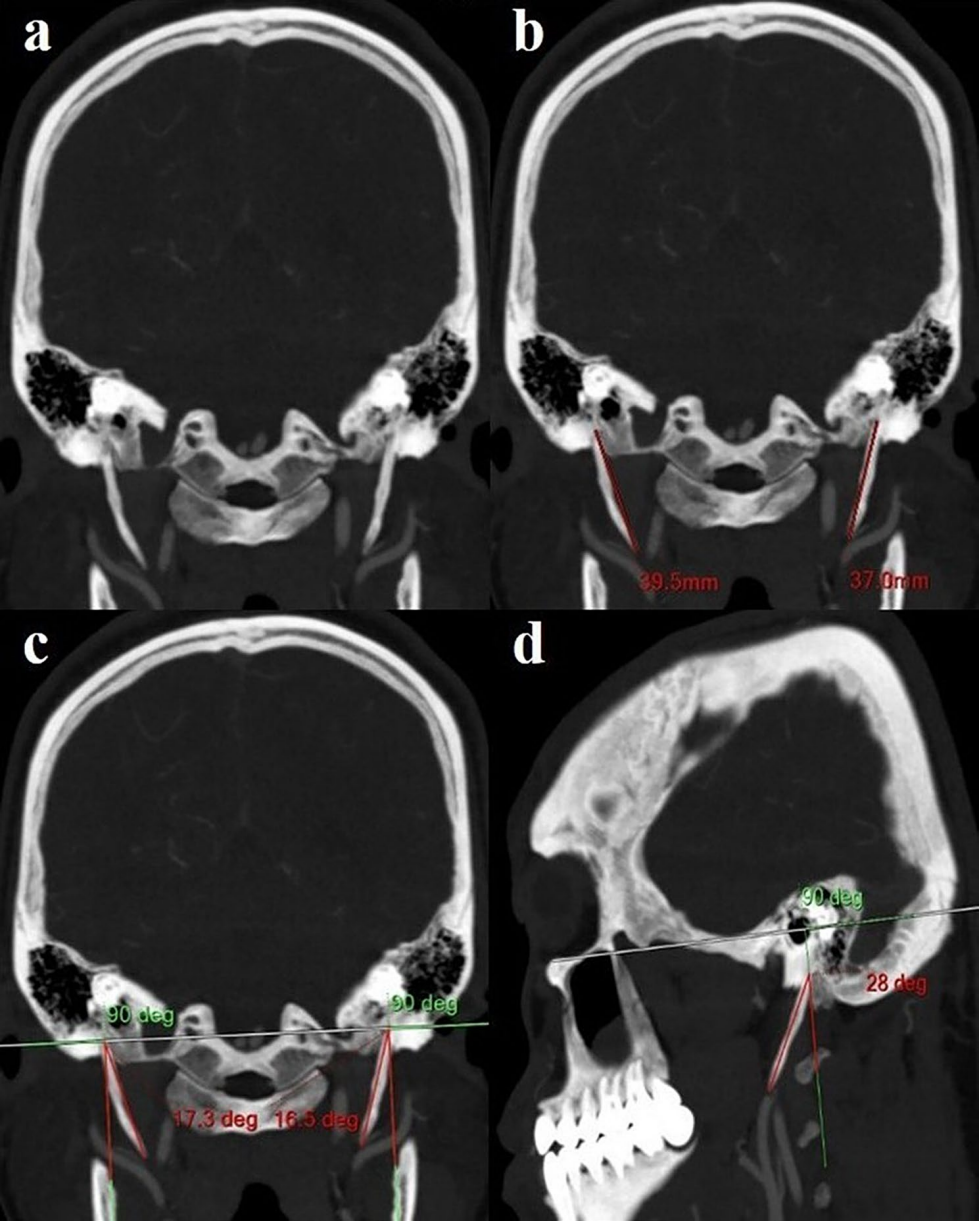
CTA-based measurements of the SP. **a** Coronal oblique MPR image showing complete visualization of both SP. **b** The red line represents the SP length, measured from the midpoint of the SP base to the tip. **c** The white line is the horizontal baseline, the green line is the vertical reference axis, and the red angle represents the medial inclination angle. **d** The white line is the OML used as the horizontal baseline, the green line is the perpendicular reference axis, and the red angle represents the anterior inclination angle

The maximum length of the SP was measured along its long axis on coronal oblique MPR images. To ensure complete visualization and measurement reproducibility, coronal oblique MIP images with a slice thickness of 8–10 mm were generated so that the entire SP was displayed on a single image (Fig. 2a). The midpoint of the SP base was taken as the proximal reference point, and the midpoint of the distal end was taken as the distal reference point. If incomplete ossification of the SP or ossification of the stylohyoid ligament was present, the ossified segment was included as part of the distal end of the SP in the measurement (Fig. 2b).

#### The SPT–ICA distance (mm)

**Fig. 3:**
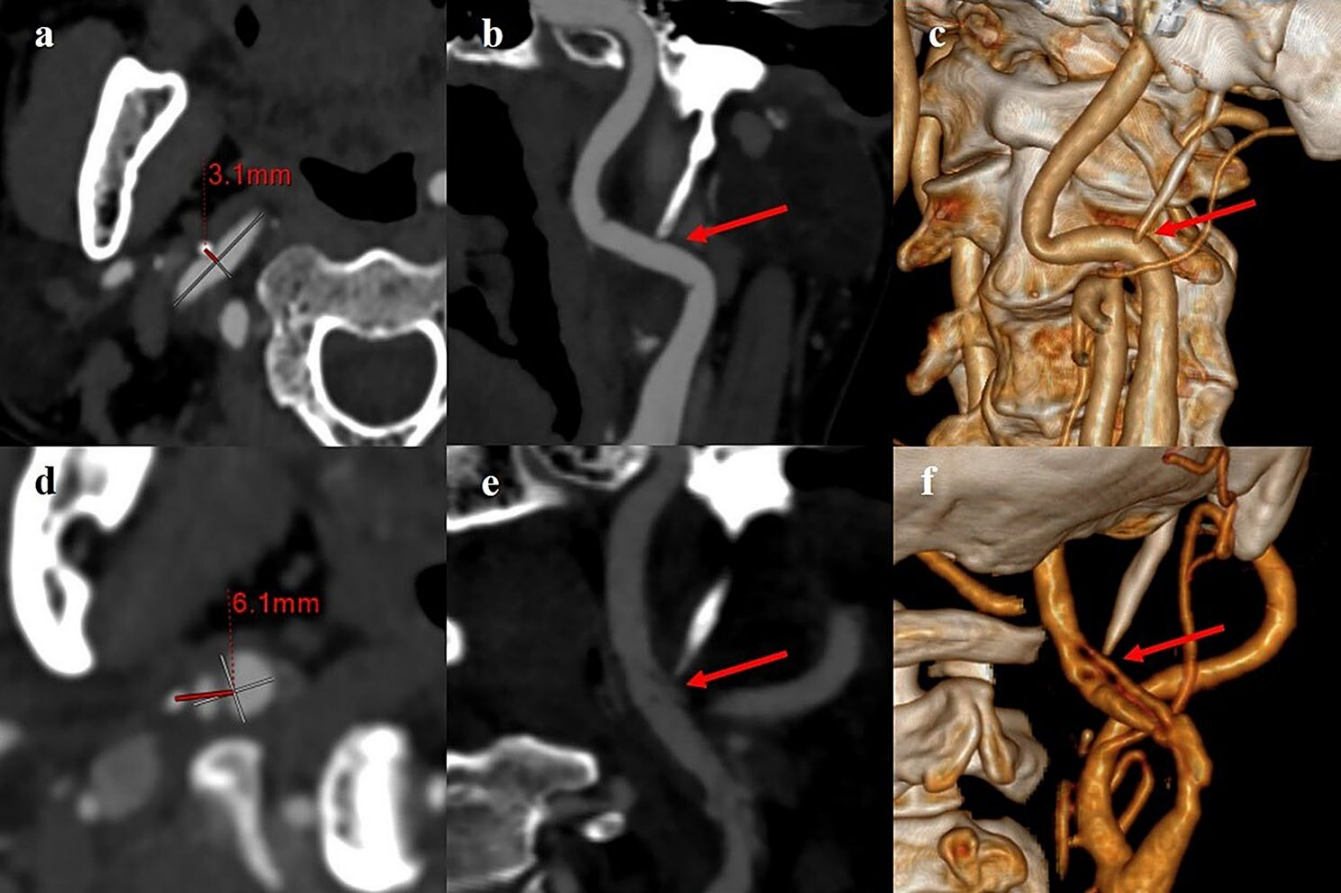
Measurement of the SPT–ICA distance and spatial relationship between the SP tip and the ICA on CTA. **a** The distance from the SP tip to the geometric center of the ICA lumen (red line). **b-c** The spatial relationship between the SP tip and the ICA in the non-dissection case (red arrow). **d** At the SP tip level, the distance is taken from the SP tip to the geometric center of the dissected-artery complex (red line). **e-f** The spatial relationship between the SP tip and the ICA in the dissection case (red arrow)

The distance from the SP tip to the geometric center of the ICA lumen was measured on axial images (Fig. 3a-c). This center-based measurement was chosen as a standardized and reproducible metric, because in dissected arteries the vessel contour may be distorted by mural hematoma, stenosis, dilatation, or partial occlusion, making the true shortest SP–ICA distance more difficult to define consistently across cases and controls [15, 16]. In the dissection group, if the location of the ICAD coincided with the level of the SP tip, the distance was measured from the midpoint of the SP tip to the geometric center of the dissected-artery complex, in order to minimize errors caused by arterial dilation or stenosis due to dissection (Fig. 3d-f). Even if the ICA was partially occluded, this point could still be reliably located on CTA. Patients in whom severe stenosis or occlusion of the ICA prevented reliable visualization and measurement of the ICA were excluded from the analysis [16].

#### The SP orientation

The medial inclination angle was measured on coronal oblique MIP images with a slice thickness of 8–10 mm, ensuring that the entire SP was fully displayed on a single image. The line connecting the midpoints of the bilateral SP bases was used as the horizontal baseline, and its perpendicular line was taken as the reference vertical axis. The angle between the long axis of the SP and this vertical axis was recorded as the medial inclination angle. If the SP was curved or segmented, its long axis was defined as the line connecting the midpoint of the base and the midpoint of the distal end (Fig. 2c).

The anterior inclination angle was measured on sagittal oblique MIP images with a slice thickness of 5–8 mm, ensuring that the target SP was fully displayed on a single image. The OML was used as the horizontal baseline, and its perpendicular line was taken as the reference vertical axis. The angle between the long axis of the SP and this vertical axis was recorded as the anterior inclination angle (Fig. 2d).

### Statistical analysis

Statistical analyses were performed using IBM SPSS Statistics (version 26.0). Continuous variables are presented as mean ± standard deviation or median (interquartile range), as appropriate, and categorical variables as n (%). Categorical variables were compared using the χ² test. Continuous variables were compared using paired-samples t tests or Wilcoxon signed-rank tests for within-subject analyses, and independent-samples t tests or Mann–Whitney U tests for between-group analyses, as appropriate. Because controls were frequency matched at the group level rather than individually paired, multivariable analyses were performed using unconditional logistic regression. Inter-observer and intra-observer agreement for styloid process (SP) measurements were evaluated using the intraclass correlation coefficient (ICC), and the mean of the two readers’ measurements was used for subsequent analyses. A multivariable logistic regression model was developed using the prespecified SP morphological parameters. The model-derived predicted probabilities were used to generate the ROC curve and calculate the AUC. Cut-points were determined by maximizing the Youden index. A two-sided p value < 0.05 was considered statistically significant.

Within the ICAD group, SP parameters on the dissected side were compared with those on the contralateral side. Within the control group, SP parameters were compared between the left and right sides. For between-group comparisons, controls were selected by frequency matching on age, sex, and laterality (side of dissection), and SP parameters on the dissected side in ICAD cases were compared with those on the corresponding matched side in controls.

## Results

A total of 85 patients with unilateral extracranial ICAD and 85 frequency-matched controls were included (Table 1). In the ICAD group, 85 dissected sides and 85 contralateral sides were available for analysis. In controls, bilateral SP measurements were available for within-group left–right comparisons, and the corresponding matched side was used for between-group analyses. The inter-observer agreement for all SP measurements was good. The ICCs ranged from 0.821 to 0.849, indicating good reliability between the two observers. Intra-observer agreement was also excellent: for observer 1, ICCs ranged from 0.904 to 0.956, and for observer 2, ICCs ranged from 0.931 to 0.980.

**Table 1:** Demographic characteristics of ICAD patients and controls

	ICAD group (*n* = 85)	Control group (*n* = 85)	*p* value
Age (years)	60 (48.5,70.0)	61 (48.5,70.0)	0.970
Male sex, n (%)	72 (84.7%)	72 (84.7%)	1

### Within-group comparisons

In the ICAD group, there were no significant differences between the dissection and non-dissection sides in SP length (37 mm vs. 36 mm, *p* = 0.537), SPT–ICA distance (6.20 mm vs. 5.90 mm, *p* = 0.780), medial inclination angle (20.25 ° vs. 19.75 °, *p* = 0.331), or anterior inclination angle (20.62 ° vs. 20.25°, *p* = 0.414). In the control group, the medial inclination angle was greater on the right side than on the left (20.48 ° vs. 18.48 °, *p* < 0.001), whereas no significant left-right differences were observed for the other parameters (Table 2).

**Table 2:** Comparison of SP parameters in ICAD and control groups

	ICAD group		Control group		*p* value		
	Dissection side (*n* = 85)	Non-dissection side (*n* = 85)	Left side (*n* = 85)	Right side (*n* = 85)	ICAD group	Controls	
SP length (mm)	37 (33,39)	36 (32,39)	32 (30,34)	32 (29,35)	0.537	0.403	
SPT–ICA distance (mm)	6.20 (4.55,8.25)	5.90 (4.35,8.65)	8.70 (7.10,12.90)	9.00 (7.15,12.35)	0.780	0.633	
Medial inclination (°)	20.25 ± 4.58	19.75 ± 4.94	18.48 ± 3.74	20.48 ± 3.89	0.331	< 0.001*	
Anterior inclination (°)	20.62 ± 4.75	20.25 ± 3.84	24.47 ± 3.45	24.29 ± 3.79	0.414	0.637	

### Between-group comparisons

**Fig. 4:**
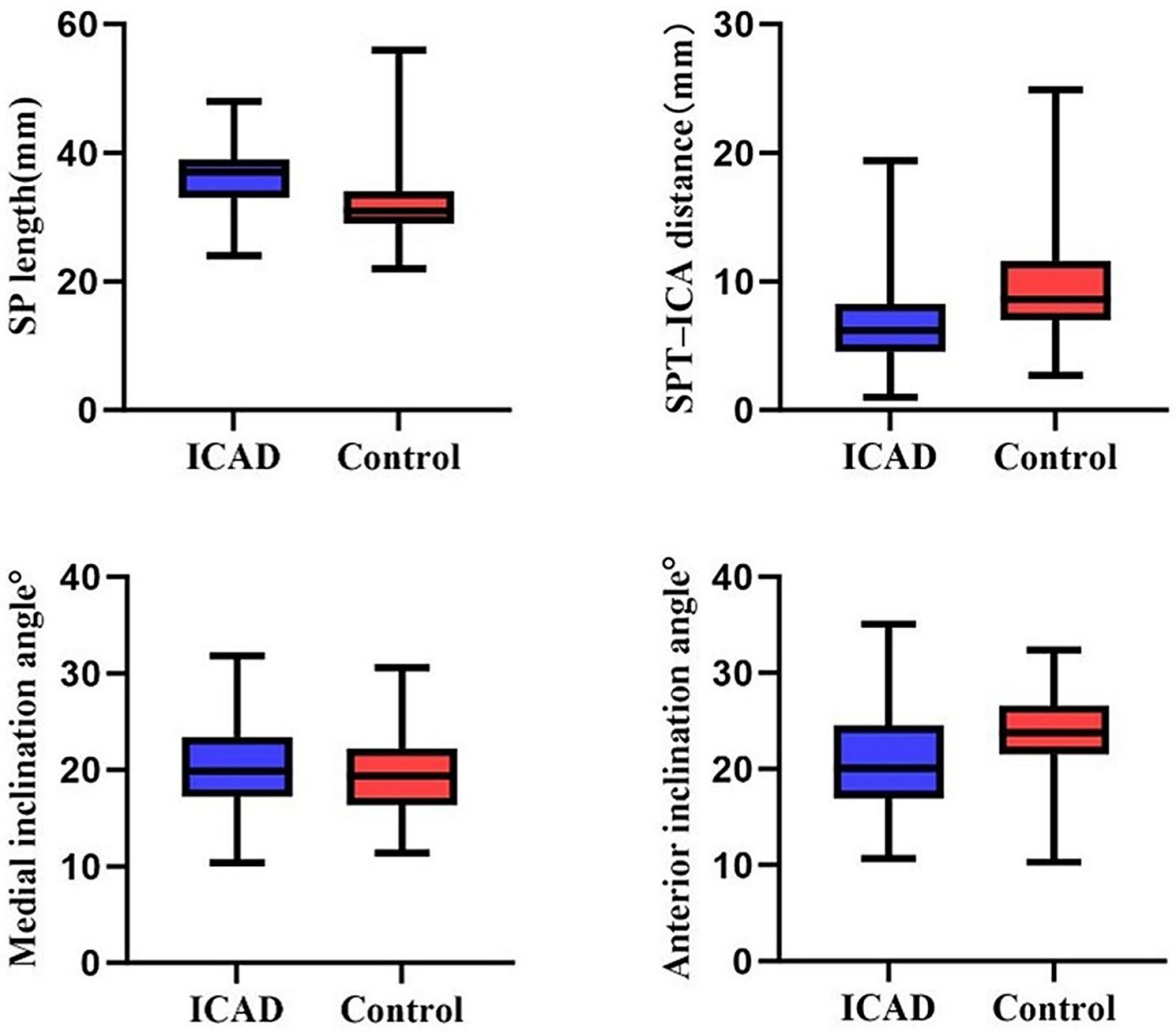
Boxplots of SP morphological parameters in the ICAD and control groups. The ICAD group showed a longer SP, a shorter SPT–ICA distance, and a smaller anterior inclination angle

In the dissection group, the SP on the dissected side was longer than that on the corresponding matched side in the control group (37 [33–39] mm vs. 31 [29–34] mm, *p* < 0.001), the SPT–ICA distance was shorter (6.20 [4.55–8.25] mm vs. 8.60 [7.00–11.60] mm, *p* < 0.001) and the anterior inclination angle was smaller on the dissection side (20.62 ± 4.75 ° vs. 24.00 ± 3.68 °, *p* < 0.001). There was no significant difference in the medial inclination angle between the two groups (20.25 ± 4.58 ° vs. 19.44 ± 3.82 °, *p* = 0.210) (Table 3; Fig. 4).

**Table 3:** Comparison of SP parameters between ICAD and control groups

	ICAD group (*n* = 85)	Control group (*n* = 85)	*p* value
SP length (mm)	37 (33,39)	31 (29,34)	< 0.001*
SPT–ICA distance (mm)	6.20 (4.55,8.25)	8.60 (7.00,11.60)	< 0.001*
Medial inclination (°)	20.25 ± 4.58	19.44 ± 3.82	0.210
Anterior inclination (°)	20.62 ± 4.75	24.00 ± 3.68	< 0.001*

### Multivariable logistic regression analysis

**Fig. 5:**
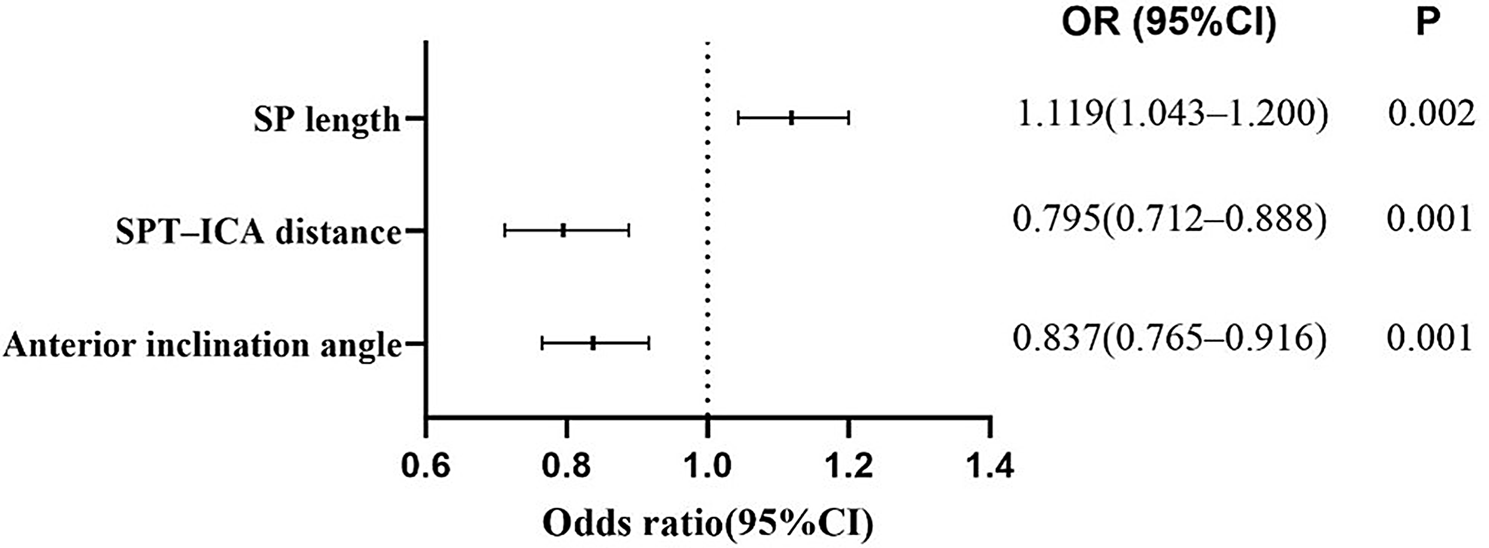
Forest plot of multivariable logistic regression analysis for ICAD. Forest plot showing OR and 95% CI of SP parameters associated with ICAD

**Fig. 6:**
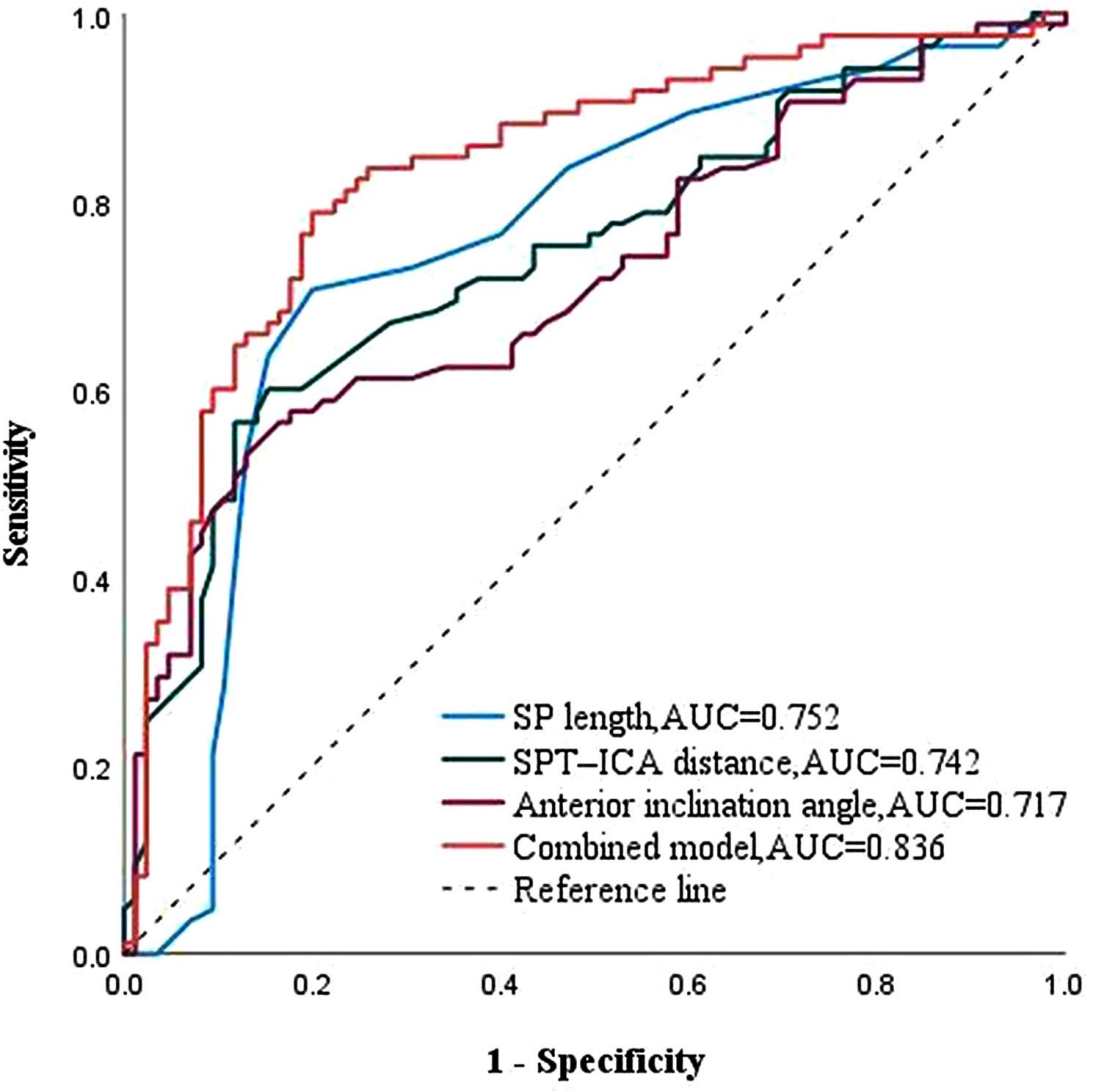
ROC curves of SP parameters for identifying ICAD. ROC curves showing the diagnostic performance of SP parameters and the combined model for identifying ICAD. The combined model showed better discrimination than each individual parameter

Multivariable logistic regression analysis showed that SP length (OR = 1.119, 95% CI 1.043–1.200, *p* = 0.002), SPT–ICA distance (OR = 0.795, 95% CI 0.712–0.888, *p* < 0.001) and anterior inclination angle (OR = 0.837, 95% CI 0.765–0.916, *p* < 0.001) were independently associated with ICAD. Longer SP length, shorter SPT–ICA distance, and smaller anterior inclination angle were associated with higher odds of ICAD. In the ROC curve analysis, the cut-point was 34.5 mm for SP length, with a sensitivity of 70.6% and a specificity of 80.0% (AUC: 0.752; 95% CI: 0.675–0.829; *p* < 0.001). The cut-point was 6.35 mm for SPT–ICA distance, with a sensitivity of 56.5% and a specificity of 88.2% (AUC: 0.742; 95% CI: 0.667–0.817; *p* < 0.001). For the anterior inclination angle, the cut-point was 20.45°, with a sensitivity of 54.1% and a specificity of 85.9% (AUC: 0.717; 95% CI: 0.640–0.795; *p* < 0.001). In ROC analysis, the combined model yielded an AUC of 0.836, outperforming each individual parameter alone (Figs. 5 and 6).

## Discussion

In this CTA-based case-control study, extracranial ICAD was associated with a distinct SP morphology characterized by an elongated SP, a shorter SPT–ICA distance, and a smaller anterior inclination angle. All three parameters remained independently associated with dissection in multivariable analysis. ROC analysis identified Youden index–optimized cut-points of 34.5 mm for SP length, 6.35 mm for SPT–ICA distance, and 20.45° for the anterior inclination angle. In addition, the combined model incorporating these three parameters showed better discriminative performance than any single parameter alone (AUC 0.836). Although SPT–ICA distance and anterior inclination angle showed relatively high specificity, their modest sensitivity suggests that these parameters should not be interpreted as standalone diagnostic markers. Taken together, these thresholds should be considered exploratory and may help standardize radiology reporting, inform future validation studies, and provide supportive morphologic evidence for a possible SP-related mechanical interaction in extracranial ICAD.

The SP length varies with the study population and measurement method [17, 18]. Imaging studies suggest an average SP length of approximately 20–25 mm [19], and most previous studies have defined an SP length greater than 30 mm as elongated [18, 20]. However, when this threshold is applied, the proportion of elongated SP reported on imaging can exceed 70%, whereas only a small fraction of these individuals develop clinical symptoms or dissection [21–23]. Systematic evidence indicates that SP elongation on imaging is far more common than clinically manifest disease [24]. In comparison with these findings, the median SP length in our control group was approximately 31 mm, slightly higher than the normal average reported in previous studies. This may be related to the fact that our control group did not consist entirely of healthy volunteers. Because the control group was derived from patients undergoing clinically indicated CTA rather than from asymptomatic healthy volunteers, indication-related selection bias cannot be excluded and may have influenced the measured SP–ICA relationship. It may also be associated with the use of CTA MPR-based measurements, as well as population differences. Conceptually, SP length provides the geometric prerequisite for potential contact, whereas SPT–ICA distance and anterior inclination angle more directly reflect spatial proximity. Together, these parameters may better capture the anatomic relationship between the SP and ICA observed in ICAD than any single metric alone.

The upper cervical region has limited anatomic reserve, and head–neck movements can alter the spatial relationship between the SP and the ICA. Previous studies have shifted focus from SP elongation to SP–ICA proximity. Raser et al. [13] reported shorter SP–ICA distances in dissection cases (4.2 mm vs. 5.6 mm, *p* = 0.02), with each 1 mm distance increase reducing risk by 21%. Renard et al. [16] reported similar findings plus frequent SP–ICA contact on the dissection side. Vascular-type Eagle syndrome-related dissection is hypothesized to stem from direct compression or sharp bony injury to the arterial wall [25, 26]. Isolated SP elongation rarely causes dissection, as risk rises only when combined with triggering factors like close proximity and head–neck movements. In this study, we measured the distance from the tip of SP to the center of the arterial lumen, or to the center of the dissected-artery complex on axial images to reduce measurement bias caused by dissection-related ICA dilatation or occlusion [15, 16]. Measurements were obtained at the level of the SP tip rather than the closest point along the shaft because the tip is a reproducible anatomic landmark on routine CTA and may be relevant to focal SP–ICA proximity. The tip is typically sharper and has a smaller contact area, which may be associated with more focal pressure or shear stress at the local interface. Additionally, during head–neck rotation, the tip likely undergoes greater displacement than the shaft, and its relationship with adjacent structures may be more dynamic, making it more likely to cause dynamic “contact–separation” alternations, thus triggering direct compression or repeated friction under specific postures. Notably, despite its anatomic relevance and relatively high specificity, the modest sensitivity of SPT–ICA distance suggests limited value as a standalone marker and supports its interpretation in combination with other SP morphologic features.

Only a limited number of studies have examined anterior and medial inclination angles, and results have been mixed. Muthusami et al. [27] found no association between SP angulation (anterior or medial) and ICAD, whereas Tardivo et al. [28] reported that the SP angles were sharper and more medially inclined in the dissection group compared to the non-dissection group (67.67 ° vs. 73.24 °, *p* = 0.002). In our study, the medial inclination angle did not differ significantly between groups, but the anterior inclination angle was smaller in the dissection group, indicating a more posteriorly oriented SP tip (by our definition) and closer spatial proximity to the ICA. It should be noted that the angle definitions used in previous studies are not equivalent to the anterior and medial inclination angles measured on multiplanar reformations in our study, making direct comparison of absolute values difficult. Nevertheless, both approaches point to a similar conclusion: changes in SP orientation alter the spatial position of the tip, making it more likely to approach the ICA. In our study, the anterior inclination angle remained independently associated with ICAD after adjustment for SP length and SPT–ICA distance, suggesting that SP orientation provides additional information beyond simple elongation and proximity. However, its modest sensitivity indicates that orientation alone may be insufficient for discrimination and is better viewed as providing complementary morphologic information rather than serving as a standalone marker.

Within-group comparisons revealed no significant differences in SP length, SPT–ICA distance and angles in the dissection group. In controls, only the medial inclination angle showed a slight difference, whereas the other parameters were broadly similar between sides, supporting symmetrical SP development in individuals. The absence of significant differences between the dissected and contralateral sides in the ICAD group suggests that SP morphology alone may not determine the laterality of dissection. Rather, these morphologic features may reflect a background anatomic predisposition, while the occurrence of dissection on one side may depend on additional local or dynamic factors. This interpretation also argues against over-reliance on static SP morphology alone in clinical decision-making. Accordingly, bilateral assessment of SP morphology may still be informative when marked elongation or close SP–ICA proximity is identified on CTA. Given the high prevalence of imaging-defined elongated SP, prophylactic styloidectomy is not recommended based solely on SP length [29]. For patients with elongated SP and close SP–ICA proximity, dynamic posture-related imaging may be clinically useful [30]. Styloidectomy could be considered for recurrent or refractory cases despite standard medical treatment [31, 32], and potential SP compression of stents should be monitored during carotid stenting [29].

This study has several limitations. First, as a single-center retrospective analysis, it is inherently susceptible to case selection bias, and some clinical data, including certain ICAD-related risk factors, may be incomplete or inconsistently documented due to the retrospective nature. Accordingly, residual confounding cannot be excluded. Second, the control group was drawn from a clinically indicated CTA population rather than from healthy volunteers, which may have limited group comparability and introduced indication-related selection bias. Third, all SP morphological measurements were conducted manually. Although both inter-observer and intra-observer agreement were good in this study, some residual measurement variability remains inevitable in manual assessments, particularly for the anterior inclination angle, which was measured using the OML as the horizontal baseline and may still be affected by head-position variability across retrospective CTA acquisitions. In future research, we plan to expand the sample size, initiate multi-center collaborations to improve generalizability, and develop a deep learning-based automated measurement model for SP parameters to enhance accuracy and efficiency. Lastly, the lack of prospective cohort data means the proposed thresholds for SP–ICA proximity and morphological risk factors remain unvalidated, and the absence of long-term follow-up prevents assessment of outcomes such as dissection recurrence or progression. Additionally, CTA was acquired in a single static supine position, which cannot directly capture the dynamic changes in SP–ICA spatial relationships induced by head–neck movements (e.g., flexion, rotation)—a key factor hypothesized to drive mechanical interaction between the two structures. Accordingly, any mechanical interpretation of our findings should be considered speculative. Larger prospective studies incorporating dynamic imaging are needed to validate these findings and clarify their clinical utility.

## Conclusion

CTA demonstrates that an elongated styloid process, a shorter SPT–ICA distance, and a smaller anterior inclination angle are independently associated with extracranial ICAD. Among these morphologic features, the combined model incorporating SP length, SPT–ICA distance, and anterior inclination angle showed the best discriminatory performance. These findings suggest that multidimensional assessment of SP morphology on CTA may help identify imaging markers associated with extracranial ICAD.

## Data Availability

De-identified data supporting the findings of this study derived from CTA and the relevant demographic variables used in the analyses will be made available upon reasonable request. Sharing of source CTA image data (e.g., DICOM files) is subject to institutional policies, ethics approval, and applicable data-protection regulations.
